# Identification of the Optimal Quantitative RT-PCR Reference Gene for Paper Mulberry (*Broussonetia papyrifera*)

**DOI:** 10.3390/cimb46100640

**Published:** 2024-09-26

**Authors:** Fangwei Zhou, Liang Xu, Congguang Shi, Fengying Wu, Shaozong Yang

**Affiliations:** Zhejiang Key Laboratory of Forest Genetics and Breeding, Zhejiang Academy of Forestry, Hangzhou 310023, China; zhoufangwei@njfu.edu.cn (F.Z.); xuliang@zjforestry.ac.cn (L.X.); shicongguang@zjforestry.ac.cn (C.S.); 18329041683@163.com (F.W.)

**Keywords:** *Broussonetia papyrifera*, real-time quantitative PCR, reference gene

## Abstract

Paper Mulberry (*Broussonetia papyrifera*) possesses medicinal, economic, and ecological significance and is extensively used for feed production, papermaking, and ecological restoration due to its ease of propagation, rapid growth rate, and strong stress resistance. The recent completion of the sequencing of the Paper Mulberry genome has prompted further research into the genetic breeding and molecular biology of this important species. A highly stable reference gene is essential to enhance the quantitative analysis of functional genes in Paper Mulberry; however, none has been identified. Accordingly, in this study, the leaves, stems, roots, petioles, young fruits, and mature fruits of Paper Mulberry plants were selected as experimental materials, and nine candidate reference genes, namely, *α-TUB1*, *α-TUB2*, *β-TUB*, *H2A*, *ACT*, *DnaJ*, *UBQ*, *CDC2*, and *TIP41*, were identified by RT-qPCR. Their stability was assessed using the geNorm, Normfinder, Delta Ct, BestKeeper, and RefFinder algorithms, identifying *ACT* and *UBQ* as showing the greatest stability. The expression of *BpMYB090*, which regulates the production of trichomes, was examined in the leaves of plants of the wild type (which have more trichomes) and mutant (which have fewer trichomes) at various developmental stages to validate the results of this study. As a result, their identification addresses a critical gap in the field of Paper Mulberry research, providing a solid foundation for future research that will concentrate on the characterization of pertinent functional genes in this economically valuable species.

## 1. Introduction

The deciduous tree or shrub, Paper Mulberry (*Broussonetia papyrifera*), belongs to the Moraceae family and is distributed over various regions of China, East Asia, Southeast Asia, and northern South Asia. It is considered a pioneering plant and a significant representative of local tree species [1,2]. Due to their rapid reproduction, growth, and remarkable resistance to stressful conditions, Paper Mulberry trees are extensively used in environmental restoration, paper manufacturing, and animal feed production. The trees contain high levels of protein (between 18 and 24% of the dry weight), similar to those of alfalfa hay and greater than those of grass or leguminous roughage. It is generally regarded as a woody feed with substantial development opportunities [3]. The bark of Paper Mulberry possesses elongated fibers with beneficial morphological characteristics, higher cellulose content, and lower lignin levels. It may be easily pulped, making it suitable for weaving or papermaking [4,5]. The tree is also a valuable pioneer in establishing or restoring plant communities. As it can reproduce both asexually through root sprouting and via the production of seeds, the rapid growth of Paper Mulberry roots and trees in damaged ecosystems is important as a means of preventing soil erosion and improving the quality of the soil microenvironment [4,5]. Paper Mulberry is extensively dispersed, highly versatile, possesses a small genome, and displays various phenotypic characteristics. Therefore, it is an exemplary model organism for a functional study of genes in woody plants. The sequenced genome of the Paper Mulberry has recently been published, allowing the application of biotechnological strategies for the genetic breeding and molecular study of this important woody plant species. 

Quantifying gene expression is necessary for research centered on gene functions. Among the techniques established for such investigations, RT-qPCR is the most commonly employed due to its superior specificity, reproducibility, and sensitivity, facilitating quantification with a high level of precision [6,7]. The accuracy of RT-qPCR is influenced by factors such as the quality of DNA and RNA, amplification efficiency, and the original sample size. As a result, reference genes are essential to ensure precision and reduce the possibility of bias in interpreting gene expression patterns [8,9]. Optimal internal reference genes exhibit stable intracellular expression without interference by external factors. Housekeeping genes are commonly used, including genes encoding actin (*ACT*), TIP41-like protein (*TIP41*), *β*-Tubulin (*β*-*TUB*), ubiquitin (*UBQ*), and 18S ribosomal RNA (*18S rRNA*) [10,11,12,13,14]. It is essential that reference genes are present across several developmental stages and tissues and that they remain unaffected by environmental influences and unidentified stressors [15]. Therefore, it is essential to identify genes that meet these criteria to ensure the most accurate assessment of gene expression. 

Reference gene expression has been extensively characterized in species including *Arabidopsis thaliana* [16], *Oryza sativa* [17], *Zingiber officinale* [18], *Liquidambar formosana* [19], *Sorghum bicolor* [20], *Citrullus lanatus* [21], *Cucumis melo* [22], *Paeonia suffruticosa* Andr. [23], *Populus* [24], *Eucalyptus* [25], and *Dendrobium huoshanense* [26]. Results from these studies suggest that, even within a given species, the most appropriate reference genes vary among tissue types or stressor conditions. In Paper Mulberry, *BpDOUB* and *BpNADH* were recognized as suitable reference genes in roots and leaves under Cd stress; however, no reference genes showing stable expression across several tissues have been discovered [27]. The instability in gene expression may lead to substantial inaccuracies in data related to different experimental settings, affecting the precision of target gene detection initiatives. A swift surge of studies has emerged regarding the functionality of Paper Mulberry genes following the completion of its entire genome sequencing [28]. Therefore, it is necessary to identify appropriate reference genes expressed across many tissues in Paper Mulberry to ensure the reliability of expression data. 

Six different Paper Mulberry tissue types (leaves, stems, roots, petioles, young fruits, and mature fruits) were selected as experimental materials. Using genomic and transcriptomic data, *ACT*, *UBQ*, *α-TUB1*, *α-TUB2*, *β-TUB*, *TIP41*, *CDC2*, *H2A*, and *DnaJ* were initially identified as reference genes through the cloning of homologous genes from the Paper Mulberry genome. The expression of these genes in the six tissue types was analyzed using RT-qPCR, and stability was assessed using the GeNorm, NormFinder, Delta Ct, and BestKeeper algorithms, followed by the geometric mean and the ReFinder network program to determine the gene combination with the greatest stability. The expression levels of the two genes demonstrating the highest and lowest stability were later employed to evaluate the expression of the *BpMYB090* gene, which regulates trichome development, in leaves at different developmental stages and in mutant plants with reduced trichome density as an additional measure of stability. The discovered genes collectively provide a crucial molecular foundation for precise RT-qPCR-based assessments of Paper Mulberry gene expression across many tissues and organs, thus underpinning functional research and transgenic breeding initiatives. 

## 2. Materials and Methods

### 2.1. Plant Materials

**Figure 1:**
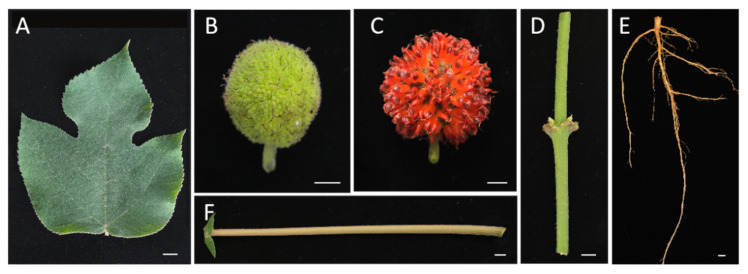
Different Paper Mulberry tissues. (**A**) leaves, (**B**) young fruit, (**C**) mature fruit, (**D**) stems, (**E**) roots, and (**F**) petioles. Scale bar: 1 cm.

Specimens of Paper Mulberry were cultivated at the Zhejiang Academy of Forestry. In August 2023, three trees of comparable height demonstrating rapid development were selected for sample collection, during which leaves, petioles, immature fruits, mature fruits, stems, and roots were systematically collected from each specimen (Figure 1). Three replicate samples per tree were harvested for each tissue type. After collection, these samples were transferred into centrifuge tubes, frozen in liquid nitrogen, and stored at −80 °C. 

### 2.2. Extraction of RNA and cDNA Synthesis

Paper Mulberry tissue samples were fully ground, after which an RNAprep Pure Polysaccharide Polyphenol Plant Total RNA Extraction Kit (TIANGEN, Nanjing, China) was used for RNA isolation. RNA integrity was analyzed via 1% agarose gel electrophoresis (AGE), and a NanoDrop™ One instrument (Thermo Fisher, Waltham, MA, USA) was used to assess RNA quality and quantity. Following this, cDNA was synthesized for each sample employing the One-Step gDNA Removal and cDNA Synthesis SuperMix kit (Trans, Nanjing, China), using a reaction volume of 20 μL and 1 μg of total RNA. RNA from individual samples was used for cDNA synthesis and subsequently stored at −20 °C.

### 2.3. Reference Gene Selection and Primer Design

In total, nine potential reference genes were chosen (*ACT*, *UBQ*, *α-TUB1*, *α-TUB2*, *β-TUB*, *TIP41*, *CDC2*, *H2A* and *DnaJ*), while *BpMYB090* was selected as a representative target gene (Table 1). Full-length and mRNA sequences for these reference genes were determined using the Paper Mulberry genome annotation file, and Primer Premier 5.0 was used for primer design with the following constraints: primer length, 18–24 bp; G.C. content, 45–55%; melting temperature, ~60 °C; and product size, 100–250 bp. The specificities of the primers were evaluated by BLAST comparisons with the Paper Mulberry genome, following which the final primers were synthesized by Shenggong Biotechnology Co., Ltd (Shanghai, China). An equal amount of the cDNA samples were combined and subjected to a five-fold dilution with ddH_2_O. Amplified PCR products were imaged following agarose gel electrophoresis, with only primers exhibiting sufficient specificity, products of the expected size, and an absence of primer dimers being selected for further use. Then, RT-qPCR was used to test the specificity of these primers. Primers must show a single melt curve peak, free of any negative control peaks or signs of impurities, to be considered appropriate for use in future experiments. These outcomes guided the selection of final primers for additional use. 

### 2.4. Reference Gene RT-qPCR Analyses 

Following the guidelines, RT-qPCR assays were conducted using a QIAquant 96 2plex equipment (QIAGEN, Hilden, Germany) and the PowerUpTM SYBR^TM^ Green Master Mix (Thermo Fisher, Waltham, MA, USA). Each reaction contained 2 μL of diluted cDNA templates, 0.2 μL of each of the forward and reverse primers (10 μM), 10 μL of PowerUp™ SYBR™ Green Master Mix, and ddH_2_O to a final 20 μL volume. Then, RT-qPCR analyses were performed as follows: 95 °C for 3 min, 40 cycles of 95 °C for 15 s, 60 °C for 15 s, and 72 °C for 30 s. A default melt curve analysis was conducted from 55–95 °C with 0.3 °C increments for 60 s, recording the corresponding fluorescent signals. Sample analyses were repeated in triplicate.

### 2.5. Construction of Standard Curves for Reference Gene Primers

To enable standard curve construction, a five-fold cDNA dilution series was prepared for each sample (dilutions: 1, 1/5, 1/25, 1/125, 1/625), with ddH_2_O serving as a negative control. As previously mentioned, RT-qPCR was used to determine the Ct values for each gene, using the QIAquant 96 2plex system. A standard curve was generated on the graph by placing log-transformed dilutions on the horizontal axis and average Ct values on the vertical axis. This allowed for the computation of the correlation coefficient (R^2^) and slope (K). Amplification efficiency (E) values for each of these reference genes were calculated as E = [5^(1/−K)^ − 1] × 100%, enabling further analyses of whether the specific primers, templates, and amplification program were suitable for RT-qPCR analyses using the following standards: R^2^ > 0.99, 90% ≤ E ≤ 110%. Analyses were repeated in triplicate to ensure reliability.

### 2.6. Data Processing

geNorm evaluates the stability values (M) for reference genes, and those with the highest expression stability can be chosen for normalization [9]. The M value is used to compare reference genes that show good stability; a cut-off value of 1.5 is used, meaning that M values less than 1.5 indicate that a reference gene is suitable, and smaller M values imply higher reference gene stability. This algorithm can also determine optimal reference gene combination numbers based on measures of the V_n/n+1_ value. When paired V_n/n+1_ < 0.15, the optimal reference gene number is n, whereas the n + 1st gene should be introduced if V_n/n+1_ > 0.15 [29].

The model-based NormFinder approach allows for the calculation of expression stability (S) values according to the 2^−ΔCt^ method [30], allowing for appropriate reference gene selection, with smaller S values indicating greater stability [31].

The ΔCt algorithm assesses gene pairs’ relative expression levels to identify suitably stable reference genes in a given sample. Consistency between ΔCt values for two genes across different samples indicates expression stability. In contrast, fluctuating ΔCt values indicate unstable expression for one or both genes. Furthermore, 3 to 5 additional genes may be integrated into the study to provide further insights. As a result, genes are categorized to identify the most suitable reference gene candidates [32].

Gene stability can be evaluated using BestKeeper, which analyses repeated paired correlations and regression based on the SDs and CVs for paired Ct values. Smaller values in these studies correspond to more stability [33].

RefFinder is an algorithm that facilitates the thorough evaluation and ranking of findings obtained from the abovementioned four techniques, allocating suitable weights to the genes and calculating the respective geometric mean values. The genes are then allocated scores, facilitating the selection of the most optimal genes [34].

### 2.7. Stability Validation

*BpMYB090* was selected as the target to confirm the potential genes’ stability for RT-qPCR applications. The expression of *BpMYB090* in Paper Mulberry was assessed using the two most stable genes (*ACT* and *UBQ*), whereas the two genes (*H2A* and *CDC2*) showing the least stability served as controls. The relative expression of *BpMYB090* was determined by the 2^−ΔΔCt^ method [35].

## 3. Results

### 3.1. Assessment of RNA Quality and Primer Specificities

The RNA yields in samples derived from the six target Paper Mulberry tissue types (Figure 1) were examined using 1% AGE and a Thermo Fisher NanoDrop^TM^ One device. AGE analyses revealed clear RNA bands in the prepared tissue extracts, with the 28S rRNA band exhibiting a brightness level ~1.5–2.0-fold greater than that of the 18S rRNA band. These total RNA samples had OD_260/280_ values between 1.80 and 2.10, which is an excellent indication of high-quality RNA that can be used for further analysis. BLAST alignments with the Paper Mulberry genome were used to identify the genes (Table 1). The cDNA was then amplified using gene-specific primers. The 1% AGE results in every case showed that the target genes had been successfully amplified, as seen by the formation of clear, distinct bands with sizes between 117 and 196 bp, consistent with the length of these amplicons (Figure S1). A reference gene standard curve was also successfully generated, revealing a reasonably minimal disparity in the linear correlation coefficients (R^2^) between the candidate and target genes, exceeding 0.99. Primer amplification efficiency (E) ranged from 98.92% to 109.48% (Table 1) and was thus within the acceptable range (90–120%). The RT-qPCR melting curve analyses for the genes revealed single peaks devoid of primer dimer activity (Figure S2). Excellent overlap between the repeat sample amplification curves and the highly specific reference gene primer amplification was observed (Figure S3). The results demonstrated that the RT-qPCR primers developed for the nine genes were appropriate. The product specificity and efficiency values were adequate for further evaluation of reference gene stability.

### 3.2. Expression of Candidate Reference Genes

**Figure 2:**
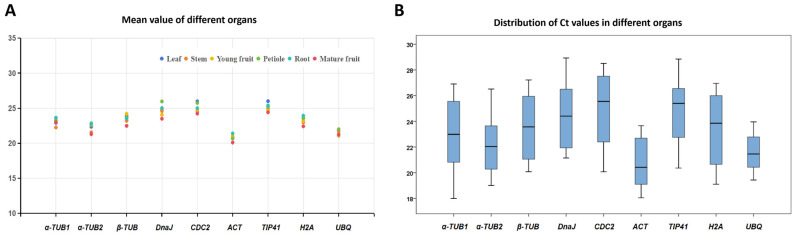
Ct values for the nine candidate reference genes in different sample types. (**A**) Average Ct values. (**B**) Distributions of Ct values in the test samples. Medians are represented by the horizontal lines, upper quartiles by the top of the boxes, and lower quartiles by the bottom of the boxes, while the upper lines represent the maximum Ct values and the outermost lines the minimum values.

Ct values show an inverse correlation with the expression levels of specific genes in designated samples. Significant variations in the Ct values of the nine genes were observed among samples (Figure 2), with average values spanning from 18.00 to 28.93 (Figure 2A), falling within the Ct value range requisite for RT-qPCR analysis. The average Ct values for the individual genes were above 20, with *H2A* exhibiting the highest expression (average Ct: 20.79) and *TIP41* exhibiting the lowest (average Ct: 25.11). Ct distributions in all test samples are shown in Figure 2B. Reduced Ct value ranges signify enhanced stability for the expression of that gene. The thorough investigations indicated that the *UBQ* gene demonstrated the least variation in expression among the reference gene candidates, ranging from 19.43 to 23.95. At the same time, the Ct values for *α-TUB1* varied most substantially, ranging from 17.99 to 26.91. The data indicate that these candidate genes have predominantly high expression levels with minimal changes, making them appropriate as reference gene candidates. However, gene levels demonstrated irregular fluctuations across several tissues, necessitating a thorough stability evaluation employing specific approaches for reference gene identification.

### 3.3. Stability Analyses 

The expression levels of candidate genes in various tissues were assessed employing the BestKeeper, geNorm, NormFinder, and ΔCt algorithms. The total gene stability in the samples was evaluated using RefFinder. 

#### 3.3.1. BestKeeper Analyses

The BestKeeper program facilitates direct analysis of Ct values derived from RT-qPCR, incorporating stability evaluations based on paired standard deviations (SD) and coefficients of variation (CV) for each value. Larger correlation coefficients and smaller CV ± SD values signify enhanced stability. A reference gene with an SD over 1 is deemed unstable. In these BestKeeper analyses, the SD values for *α-TUB1*, *β-TUB*, *CDC2*, and *H2A* were all found to exceed 1 because they were considered unstable reference genes. *UBQ* and *ACT* exhibited the highest level of stability, and the rank order of the genes in terms of CV and SD values was *UBQ* > *ACT* > *α-TUB2* > *DnaJ* > *TIP41* > *H2A* > *CDC2* > *β-TUB* > *α-TUB1* (Table 2).

#### 3.3.2. geNorm Analyses

**Figure 3:**
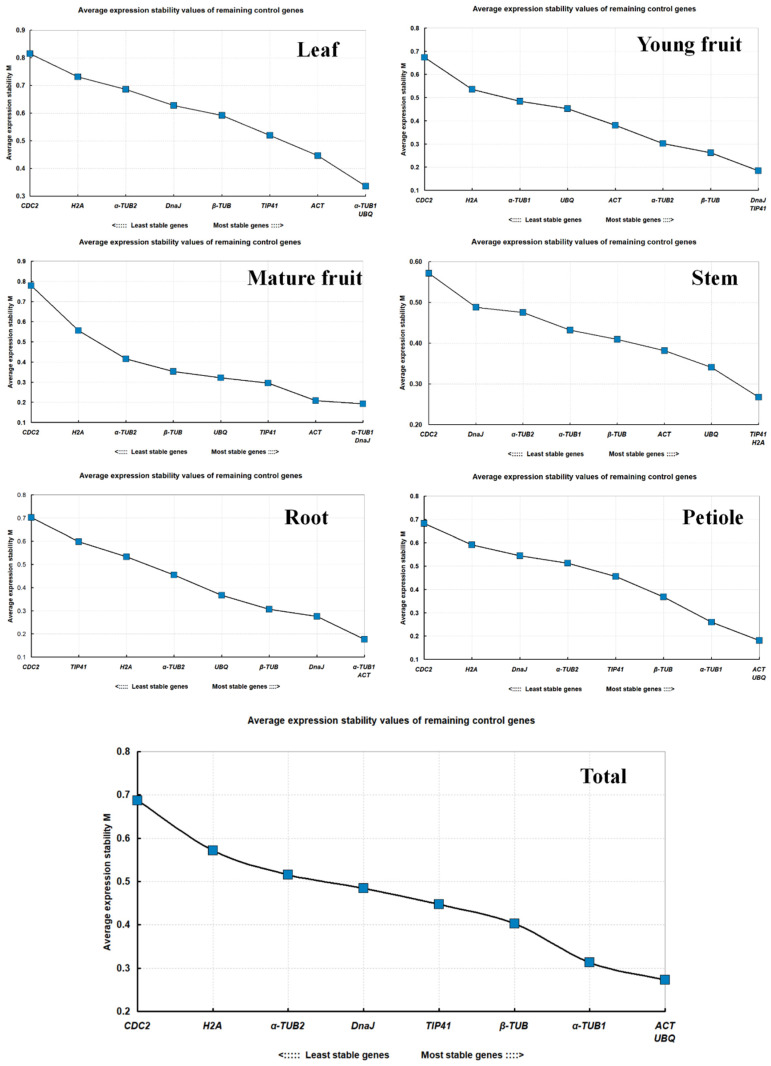
The M values for the nine candidate reference genes were analyzed using the geNorm algorithm on leaves, stems, roots, young fruits, mature fruits, petioles, and overall samples.

The geNorm program was used to evaluate the genes’ stable expression values (M). A negative association between M and stability is present when M is less than 1.5, indicating that higher M values correspond to reduced gene stability levels. Genes with an M value less than 0.5 demonstrate promise as reference genes. The M values were all less than 1.5. It is important to note that *ACT*, *UBQ*, and *α-TUB1* exhibited M values of less than 0.5 in all tissue types, indicating high stability (Figure 3). On the other hand, *CDC2* exhibited the least stable expression. The number of genes with M < 0.5 was only three in leaves, but it increased to 8 in stems, underscoring the extent to which gene stability depends upon tissue type (Figure 3). 

**Figure 4:**
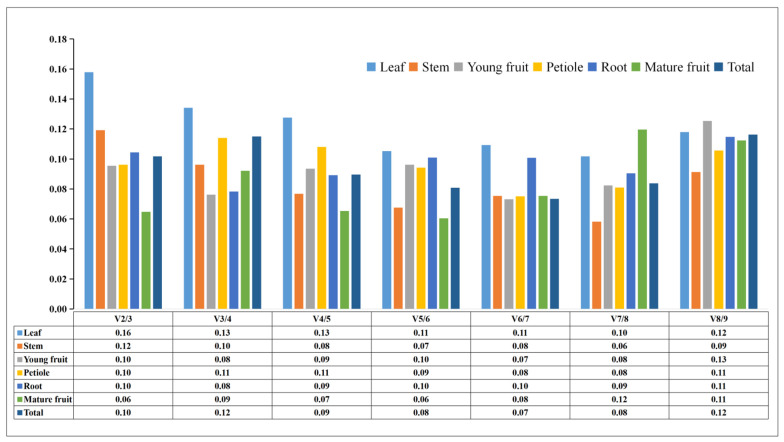
Optimal reference gene numbers, assessed by paired variation analyses (V_n/n+1_). The V_n/n+1_ values were determined using geNorm using a critical V_n/n+1_ value of 0.15, where n represents the optimal number of references.

The geNorm version 2003 software can be applied to find the optimal numbers of references using the values of paired variation (V_n/n+1_) for different tissues when V_n/n+1_ < 0.15, n represents the ideal number of references without needing n+1 references. When analyzing Paper Mulberry leaves, V_2/3_ = 0.16 > 0.15, while when a third gene was introduced, V_3/4_ = 0.13 < 0.15, demonstrating the need for accurate normalization of these genes, with *α-TUB1*, *UBQ*, and *ACT* showing the greatest stability in the leaves. In contrast, V_2/3_ = 0.12 < 0.15 in stems, consistent with the necessity of only two reference genes. TIP41 and H2A exhibit the highest stability. The optimal value of the two genes was indicated by the V_2/3_ values in roots, immature fruits, mature fruits, and petioles, which were all less than 0.15. These comprehensive analyses of reference genes across Paper Mulberry tissue types revealed *ACT* and *UBQ* to be the most stable, with the stability ranking being *ACT* = *UBQ* > *α-TUB1* > *β-TUB* > *TIP41* > *DnaJ* > *α-TUB2* > *H2A* > *CDC2* (Figure 4).

#### 3.3.3. NormFinder Analysis

NormFinder computes stability (S) values using the 2^−ΔΔCt^ method, thereby identifying the optimal reference gene with the highest stability, with higher S values indicating greater instability. The nine candidate genes showed variations in instability. The most stable components of the plant were the leaves, roots, and mature fruits, which *ACT* exhibited. *UBQ* was the most stable component of the plant in the petioles and stems. Overall, *ACT* demonstrated the greatest stability. The stability ranking was *ACT* > *UBQ* > *DnaJ* > *β-TUB* >*TIP41* > *H2A* > *α-TUB2* > *α-TUB1* > *CDC2* (Table 3).

#### 3.3.4. ΔCt Analysis

Table 4 lists the nine potential genes based on their stably expressed levels in various Paper Mulberry tissues, as determined by the ΔCt technique. *ACT* was the most stable in leaves, early fruits, mature fruits, and all samples. *DnaJ* and *UBQ* were the most stable in stems, roots, and petioles, and *β-TUB* was the second most stable in the three samples. For both individual and overall sample analysis, *CDC2* was the least stable. In ΔCt analyses, the ranking was *ACT* > *UBQ* > *DnaJ* > *β-TUB* > *TIP41* > *α-TUB2* > *α-TUB1* > *H2A* > *CDC2*. Thus, *ACT* and *UBQ* performed the best as references.

#### 3.3.5. RefFinder Analysis

**Figure 5:**
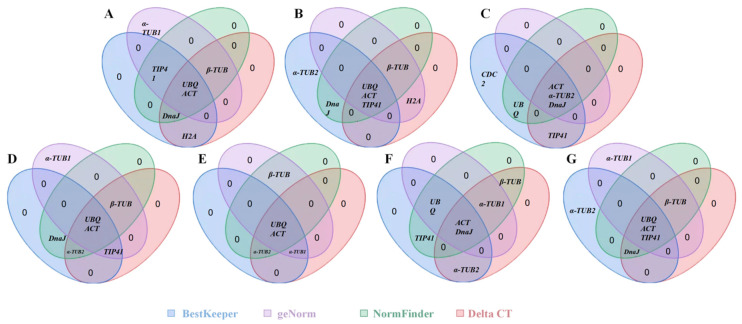
Venn diagrams showing the five reference genes according to the BestKeeper, geNorm, NormFinder, and Delta Ct analyses. Blue, purple, green, and pink ovals, respectively, indicate the five genes with the greatest stability in samples from (**A**) leaves, (**B**) stems, (**C**) young fruits, (**D**) petioles, (**E**) roots, (**F**) mature fruits, and (**G**) all samples. Genes in overlapping regions of these diagrams were identified as being among the five most stable according to more than one of the utilized algorithms.

Gene stability rankings varied in proportion to the differences in implementation between the four techniques used above. Therefore, the geometric means of the algorithm outputs were determined using the RefFinder tool. This showed that the best combination was that of *ACT* and *UBQ* for analyzing stem, leaf, and petiole samples, whereas *ACT* and *DnaJ* were the best combination in young and mature fruits, and *ACT* and *α-TUB1* were the best in root samples. When assessing all available samples, *ACT* and *UBQ* exhibited the highest levels of stability (Table 5). Figure 5 shows that, in at least three algorithms, both genes exhibited notable consistency among the top five rankings. Furthermore, it was discovered that the candidates with the lowest stability were *H2A* and *CDC2*.

### 3.4. Verification of Reference Gene Stability

**Figure 6:**
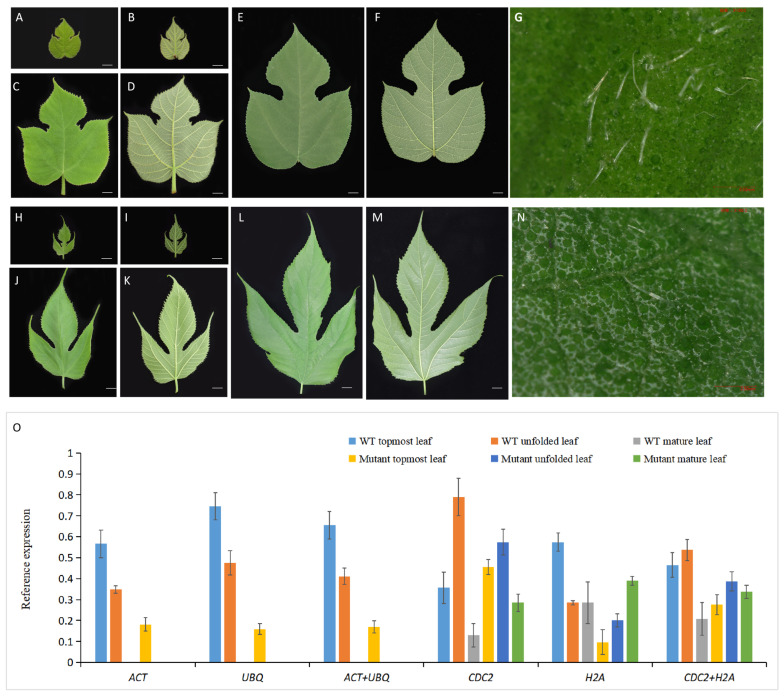
Patterns of *BpMYB090* expression. (**A**–**F**) The front and back of the topmost unfolded and mature leaves of wild-type Paper Mulberry; (**G**) Microscopic observation of trichomes in the topmost leaves of wild-type Paper Mulberry (Magnification: ×100.0); (**H**–**M**) The front and back of the topmost, unfolded, and mature leaves of the mutant Paper Mulberry; (**N**) Microscopic observation of trichomes in the topmost leaves of the mutant Paper Mulberry (Magnification: ×100.0); (**O**) RT-qPCR was used to assess *BpMYB090* expression in different Paper Mulberry tissues, normalizing gene expression using *ACT*, *UBQ*, or a combination of the two. Genes with the lowest stability, *H2A*, and *CDC2*, were also included as a comparison. Gene expression was analyzed via the. 2^−ΔΔCt^ approach.

*BpMYB090* was selected as a representative target to confirm the use of the suggested reference genes. The homologous gene of *BpMYB090* has been shown to have functional conservation and control the formation of trichomes in various species [36,37,38,39,40]. Its expression patterns were verified in leaves at different developmental stages in wild-type (containing more trichomes) (Figure 6A–G) and mutant (with fewer trichomes) (Figure 6H–N) Paper Mulberry, including topmost, unfolded, and mature leaves. Relative *BpMYB090* levels were normalized against *ACT* and *UBQ*, individually or in combination, and the results revealed consistent expression trends. *BpMYB090* was highly expressed in the upper leaves early in the trichome growth process. The trichome density gradually decreases as the leaves unfold, and *BpMYB090* expression declines. No expression of the *BpMYB090* gene was detected in mature leaves.

*BpMYB090* is involved in the development of leaf trichomes, as evidenced by the very low expression observed in the uppermost leaves of the mutant with few trichomes and the absence of expression in unfolded and mature leaves. However, when *H2A* and *CDC2* were used for normalization, the results were inconsistent and exhibited marked deviations (Figure 6O). As a result, these findings validated the stability of *ACT* and *UBQ* genes in RT-qPCR analysis, providing a foundation for further research on Paper Mulberry genes.

## 4. Discussion

Paper Mulberry wood and bark are raw materials in papermaking, while the leaves represent a protein-rich feed source [41,42]. Paper Mulberry seeds, stems, leaves, roots, and fruits also contain high flavonoid concentrations and are often employed in medicinal applications [43,44,45]. It is an ideal tree species for tailings repair and ecological restoration initiatives. Inadequate crude protein presents a significant challenge in animal husbandry, whereas ecological restoration is a worldwide concern [46,47]. Paper Mulberry-related research efforts have thus shifted to molecular biology-based analyses aimed at understanding the unique physiological characteristics of this important woody plant species. Stably expressed reference genes must be identified to facilitate data standardization and ensure the validity of quantitative analyses in this setting. There are currently few reference genes available in Paper Mulberry and other woody plant species, and no reference genes that exhibit consistent expression under various conditions have been found [48,49]. Thus, it is vital for future research that systematic efforts be used to identify appropriate reference genes.

This study used RT-qPCR to assess the stable expression of nine potential genes in six different types of Paper Mulberry tissue using the BestKeeper, geNorm, NormFinder, and ΔCt algorithms. These analyses revealed slight variations in the algorithms’ assessments of stability. While *ACT* showed the greatest stability in all four instances, BestKeeper revealed that *UBQ* was relatively stably expressed. Even greater variations were observed among these four algorithms when assessing individual tissue types. For example, BestKeeper found that *UBQ* showed the greatest stability in leaf samples, while geNorm instead identified *α-TUB1*, followed by *UBQ*. Even with these variations, however, a large degree of overlap was observed, and these findings were in line with those from prior reports identifying reference genes in *Populus euphratica* [13], *Salix suchowensis* [50], *Eucalyptus camaldulensis* [51], and other plant species. GeNorm and NormFinder showed comparable rankings for these candidate genes across all samples when comparing the overall expression stability rankings for these genes. RefFinder was used to compute geometric means from the results of the four algorithms and is applied for the thorough evaluation of stability in reference genes [52,53]. Based on the abovementioned analyses, *ACT* and *UBQ* were ultimately the best combination when analyzing Paper Mulberry samples. 

Software-based analyses alone cannot ensure the stable expression of reference genes, highlighting the necessity to validate this stability across various tissues by examining target genes of interest. Due to their elevated crude protein levels, paper mulberry leaves are extensively utilized as woody feed [54]. The elevated trichome density leads to poor palatability and reduced digestion and absorption when employed as animal feed, resulting in low feed consumption efficiency. Therefore, the research concentrates on the selective breeding of Paper Mulberry, which lacks trichomes. This study identified the *BpMYB090* gene, which regulates the growth of leaf trichomes, as the target gene for assessing reference gene stability. It was found that, when *ACT* and *UBQ* were used for standardization, *BpMYB090* levels were consistent, with high expression in the topmost leaves of the wild-type Paper Mulberry that contain high trichome densities. The trichomes matured, the density dropped, and the expression of *BpMYB090* reduced as the leaves unfolded. In the highest leaves of the Paper Mulberry mutant, which has very few trichomes, the expression of *BpMYB090* is minimal. Future research on the gene may find that its expression pattern, which gradually reduced as the trichome density dropped, was similar to the mutant phenotype of *BpMYB090*. In contrast, using *H2A* and *CDC2*, the two least stably expressed reference gene candidates, resulted in aberrant *BpMYB090* expression patterns. Thus, these findings add to an existing body of evidence supporting the validity of employing the *ACT* and *UBQ* reference gene combination in analyzing various Paper Mulberry tissues. 

*ACT* and *UBQ* were found in this investigation to be reference genes for various Paper Mulberry tissues. This is the first report published on reference genes for stable expression in different Paper Mulberry tissues. These data establish an evidence-based platform for future quantitative research on these functional genes and support the development of Paper Mulberry agronomic characteristics through advanced scientific and technological approaches. In addition, the data also provide a reference framework for selecting reference genes for Paper Mulberry-related species such as *Broussonetia kaempferi* var. australis, *Broussonetia kazinoki* Sieb.et Zucc., and *Broussonetia monoica* Hance. Although homologous reference genes typically exhibit conservation in closely related species, differences in expression stability between species are also evident. As a result, a thorough study incorporating several reference gene types is necessary for the precise assessment of target gene expression and the quantitative analysis outcomes across diverse species.

## 5. Conclusions

In the present study, nine putative reference genes were expressed in Paper Mulberry using RT-qPCR, and their stability was assessed using five distinct statistical algorithms. We conclude that *ACT* and *UBQ* are the most suitable RT-qPCR reference genes for various tissues of Paper Mulberry, and *BpMYB090* was used as a target gene to validate the stability of the selected reference genes. To our knowledge, the current research is the first to discover suitable reference genes for normalizing gene expression analyses via RT-qPCR in several tissues of Paper Mulberry. This establishes the basis for gene expression and associated molecular biology research on Paper Mulberry. 

## Figures and Tables

**Table 1:** Candidate reference genes, their primer sequences, and amplification by RT-qPCR.

Gene	Gene Description	Gene ID	Primer Sequence F/R(5′-3′)	Product Size (bp)	Efficiency (%)	R^2^
*α-TUB1*	Alpha-tubulin1	Bp02g1361.1	GGTGGAGCCATACAACAGT	178	98.92	0.9951
			GGCAGTGAGCGATGAGAT			
*α-TUB2*	Alpha-tubulin2	Bp11g0006.1	CACTGGACTACAAGGGTTT	138	102.45	0.9971
			GGTGAAGGGTAGATGGTG			
*β-TUB*	β-tubulin	Bp06g0215.1	CGGATGATGCTCACCTTCTC	117	105.83	0.9947
			CATACACTCGTCGGCGTTCT			
*TIP41*	Type 2A phosphatase activator	Bp11g1240.1	TGCTTATGAGACTAAGGGAC	136	107.77	0.9955
			GCGGAATCAGTAGGGTAT			
*ACT*	Actin	Bp01g3225.1	AATGGTGAAGGCTGGGTT	178	101.28	0.9954
			ACCGTGCTCAATGGGATA			
*CDC2*	Cyclin dependent kinase-putative	Bp07g1967.1	GGCATTGCTTACTGTCATTC	175	106.73	0.9913
			GTGCTCTGTACCAAAGGGTC			
*H2A*	Histone H2A	Bp05g0572.1	TGGCTGCTGAGGTGCTAGAGTT	174	107.61	0.9961
			GGAGGAGGTTGTGGATGTTGG			
*DnaJ*	Chaperone protein DnaJ 49	Bp05g0847.1	AGAGGCGAACCACGAGACAT	196	109.48	0.9920
			ACATTGAACCCACCAGACCC			
*UBQ*	Ubiquitin family	Bp10g1600.1	CCCTCGCCGACTACAACA	189	109.12	0.9955
			TCAGCCTCTGGACCTTGC			
**Target gene**						
*BpMYB090*	MYB	Bp12g0904.1	TACCTGACGGCTTGGCTAC	185	103.21	0.9942
			ATCCTCAATCCACCGCTCT			

**Table 2:** BestKeeper calculations of SDs and CVs corresponding to Ct values for the specific genes.

	*α-TUB1*	*α-TUB2*	*β-TUB*	*DnaJ*	*ACT*	*TIP41*	*H2A*	*UBQ*	*CDC2*
**Leaf**									
**SD**	**1.51**	**1.51**	**1.12**	**0.74**	**0.64**	**0.79**	**0.96**	**0.57**	**1.74**
**CV**	**6.22**	**2.53**	**5.52**	**3.2**	**3.4**	**3.58**	**4.28**	**2.72**	**5.15**
**Stem**									
**SD**	**2**	**0.8**	**2**	**0.95**	**0.73**	**1**	**1.53**	**0.61**	**1.95**
**CV**	**7.32**	**3.6**	**7.45**	**3.75**	**3.38**	**3.73**	**5.78**	**2.63**	**6.99**
**Young fruit**									
**SD**	**1.96**	**0.87**	**1.91**	**0.95**	**0.82**	**1.07**	**1.46**	**0.69**	**0.94**
**CV**	**7.08**	**3.87**	**6.93**	**3.82**	**3.77**	**3.96**	**5.5**	**2.99**	**3.94**
**Petiole**									
**SD**	**2.34**	**0.77**	**1.07**	**0.75**	**0.77**	**0.83**	**1.54**	**0.6**	**1.31**
**CV**	**8.47**	**3.48**	**4.53**	**3.08**	**3.57**	**3.12**	**5.71**	**2.55**	**4.54**
**Root**									
**SD**	**0.83**	**0.58**	**1.32**	**0.3**	**0.48**	**0.89**	**1.12**	**0.58**	**1.62**
**CV**	**2.81**	**2.59**	**4.63**	**1.21**	**2.2**	**3.35**	**4.12**	**2.44**	**5.67**
**Mature fruit**									
**SD**	**1.32**	**0.75**	**2**	**0.89**	**0.6**	**1.12**	**1.56**	**0.49**	**1.3**
**CV**	**5.09**	**3.4**	**7.54**	**3.59**	**2.75**	**4.19**	**5.9**	**2.07**	**5.82**
**Total**									
**SD**	**2.01**	**0.73**	**1.93**	**0.8**	**0.7**	**0.98**	**1.43**	**0.64**	**1.78**
**CV**	**7.26**	**3.26**	**7.05**	**3.25**	**3.21**	**3.66**	**5.39**	**2.74**	**6.27**

Deeper green coloration is indicative of higher levels of stability.

**Table 3:** Determination of the S values of the 9 candidate genes using NormFinder.

Rank		1	2	3	4	5	6	7	8	9
Leaf	Gene	*ACT*	*UBQ*	*β-TUB*	*DnaJ*	*TIP41*	*H2A*	*α-TUB2*	*α-TUB1*	*CDC2*
	stability	0.119	0.120	0.317	0.375	0.418	0.446	0.493	0.541	0.723
Stem	Gene	*UBQ*	*ACT*	*DnaJ*	*TIP41*	*β-TUB*	*α-TUB1*	*α-TUB2*	*H2A*	*CDC2*
	stability	0.095	0.178	0.203	0.226	0.230	0.281	0.343	0.347	0.562
Young fruit	Gene	*DnaJ*	*UBQ*	*ACT*	*β-TUB*	*α-TUB2*	*H2A*	*α-TUB1*	*TIP41*	*CDC2*
	stability	0.064	0.064	0.107	0.216	0.305	0.333	0.445	0.450	0.776
Petiole	Gene	*UBQ*	*DnaJ*	*ACT*	*β-TUB*	*α-TUB2*	*TIP41*	*H2A*	*α-TUB1*	*CDC2*
	stability	0.217	0.227	0.239	0.275	0.310	0.323	0.350	0.420	0.650
Root	Gene	*ACT*	*UBQ*	*DnaJ*	*β-TUB*	*α-TUB2*	*TIP41*	*H2A*	*α-TUB1*	*CDC2*
	stability	0.217	0.227	0.239	0.275	0.310	0.323	0.350	0.420	0.650
Mature fruit	Gene	*ACT*	*UBQ*	*DnaJ*	*α-TUB1*	*β-TUB*	*TIP41*	*α-TUB2*	*H2A*	*CDC2*
	stability	0.110	0.123	0.260	0.314	0.334	0.349	0.364	0.495	1.070
Total	Gene	*ACT*	*UBQ*	*DnaJ*	*β-TUB*	*TIP41*	*H2A*	*α-TUB2*	*α-TUB1*	*CDC2*
	stability	0.133	0.229	0.287	0.291	0.304	0.332	0.337	0.351	0.718

**Table 4:** ΔCt analyses of gene average STEDV for the 9 candidate genes.

Rank		1	2	3	4	5	6	7	8	9
Leaf	Gene	*DnaJ*	*β-TUB*	*UBQ*	*ACT*	*H2A*	*TIP41*	*α-TUB2*	*α-TUB1*	*CDC2*
	STEDV	0.64	0.65	0.73	0.77	0.82	0.83	0.86	0.9	1.11
Stem	Gene	*ACT*	*β-TUB*	*H2A*	*UBQ*	*TIP41*	*α-TUB1*	*α-TUB2*	*DnaJ*	*CDC2*
	STEDV	0.47	0.48	0.51	0.52	0.53	0.57	0.6	0.6	0.86
Young fruit	Gene	*ACT*	*DnaJ*	*α-TUB2*	*β-TUB*	*TIP41*	*H2A*	*UBQ*	*α-TUB1*	*CDC2*
	STEDV	0.5	0.52	0.55	0.58	0.61	0.68	0.73	0.74	1.15
Petiole	Gene	*UBQ*	*β-TUB*	*α-TUB2*	*TIP41*	*ACT*	*α-TUB1*	*H2A*	*DnaJ*	*CDC2*
	STEDV	0.59	0.59	0.61	0.62	0.64	0.64	0.71	0.75	1.01
Root	Gene	*UBQ*	*DnaJ*	*α-TUB1*	*ACT*	*α-TUB2*	*β-TUB*	*H2A*	*TIP41*	*CDC2*
	STEDV	0.55	0.55	0.57	0.61	0.63	0.72	0.75	0.87	1.07
Mature fruit	Gene	*ACT*	*β-TUB*	*α-TUB1*	*DnaJ*	*α-TUB2*	*TIP41*	*UBQ*	*H2A*	*CDC2*
	STEDV	0.6	0.58	0.65	0.65	0.65	0.68	0.68	0.95	1.56
Total	Gene	*ACT*	*UBQ*	*DnaJ*	*β-TUB*	*TIP41*	*α-TUB2*	*α-TUB1*	*H2A*	*CDC2*
	STEDV	0.63	0.58	0.64	0.67	0.68	0.69	0.70	0.75	1.13

**Table 5:** Comprehensive RefFinder rankings of the stability of the candidate genes.

Method	1	2	3	4	5	6	7	8	9
**Ranking order in Leaf (Better–Good–Average)**									
BestKeeper	*UBQ*	*ACT*	*DnaJ*	*TIP41*	*H2A*	*β-TUB*	*α-TUB1*	*α-TUB2*	*CDC2*
geNorm	*α-TUB1/* *UBQ*		*ACT*	*TIP41*	*β-TUB*	*DnaJ*	*α-TUB2*	*H2A*	*CDC2*
NormFinder	*ACT*	*UBQ*	*β-TUB*	*DnaJ*	*TIP41*	*H2A*	*α-TUB2*	*α-TUB1*	*CDC2*
Delta CT	*DnaJ*	*β-TUB*	*UBQ*	*ACT*	*H2A*	*TIP41*	*α-TUB2*	*α-TUB1*	*CDC2*
RefFinder	*UBQ*	*ACT*	*DnaJ*	*β-TUB*	*TIP41*	*α-TUB1*	*H2A*	*α-TUB2*	*CDC2*
**Ranking order in Stem (Better–Good–Average)**									
BestKeeper	*UBQ*	*ACT*	*α-TUB2*	*DnaJ*	*TIP41*	*H2A*	*CDC2*	*β-TUB*	*α-TUB1*
geNorm	*TIP41/* *H2A*		*UBQ*	*ACT*	*β-TUB*	*α-TUB1*	*α-TUB2*	*DnaJ*	*CDC2*
NormFinder	*UBQ*	*ACT*	*DnaJ*	*TIP41*	*β-TUB*	*α-TUB1*	*α-TUB2*	*H2A*	*CDC2*
Delta CT	*ACT*	*β-TUB*	*H2A*	*UBQ*	*TIP41*	*α-TUB1*	*α-TUB2*	*DnaJ*	*CDC2*
RefFinder	*UBQ*	*ACT*	*TIP41*	*DnaJ*	*β-TUB*	*H2A*	*α-TUB1*	*α-TUB2*	*CDC2*
**Ranking order in Young Fruit (Better–Good–Average)**									
BestKeeper	*UBQ*	*ACT*	*α-TUB2*	*CDC2*	*DnaJ*	*TIP41*	*H2A*	*β-TUB*	*α-TUB1*
geNorm	*DnaJ/* *TIP41*		*β-TUB*	*α-TUB2*	*ACT*	*UBQ*	*α-TUB1*	*H2A*	*CDC2*
NormFinder	*DnaJ*	*UBQ*	*ACT*	*β-TUB*	*α-TUB2*	*H2A*	*α-TUB1*	*TIP41*	*CDC2*
Delta CT	*ACT*	*DnaJ*	*α-TUB2*	*β-TUB*	*TIP41*	*H2A*	*UBQ*	*α-TUB1*	*CDC2*
RefFinder	*DnaJ*	*ACT*	*UBQ*	*β-TUB*	*α-TUB2*	*TIP41*	*H2A*	*α-TUB1*	*CDC2*
**Ranking order in Petiole (Better–Good–Average)**									
BestKeeper	*UBQ*	*DnaJ*	*α-TUB2*	*ACT*	*TIP41*	*β-TUB*	*CDC2*	*H2A*	*α-TUB1*
geNorm	*ACT/* *UBQ*		*α-TUB1*	*β-TUB*	*TIP41*	*α-TUB2*	*DnaJ*	*H2A*	*CDC2*
NormFinder	*UBQ*	*DnaJ*	*ACT*	*β-TUB*	*α-TUB2*	*TIP41*	*H2A*	*α-TUB1*	*CDC2*
Delta CT	*UBQ*	*β-TUB*	*α-TUB2*	*TIP41*	*ACT*	*α-TUB1*	*H2A*	*DnaJ*	*CDC2*
RefFinder	*UBQ*	*ACT*	*DnaJ*	*β-TUB*	*α-TUB2*	*TIP41*	*α-TUB1*	*H2A*	*CDC2*
**Ranking order in Root (Better–Good–Average)**									
BestKeeper	*α-TUB1*	*DnaJ*	*ACT*	*α-TUB2*	*UBQ*	*TIP41*	*H2A*	*β-TUB*	*CDC2*
geNorm	*α-TUB1/* *ACT*		*DnaJ*	*β-TUB*	*UBQ*	*α-TUB2*	*H2A*	*TIP41*	*CDC2*
NormFinder	*ACT*	*UBQ*	*DnaJ*	*β-TUB*	*α-TUB2*	*TIP41*	*H2A*	*α-TUB1*	*CDC2*
Delta CT	*UBQ*	*DnaJ*	*α-TUB1*	*ACT*	*α-TUB2*	*β-TUB*	*H2A*	*TIP41*	*CDC2*
RefFinder	*α-TUB1*	*ACT*	*DnaJ*	*UBQ*	*β-TUB*	*α-TUB2*	*H2A*	*TIP41*	*CDC2*
**Ranking order in Mature Fruit (Better–Good–Average)**									
BestKeeper	*UBQ*	*ACT*	*α-TUB2*	*DnaJ*	*TIP41*	*CDC2*	*α-TUB1*	*H2A*	*β-TUB*
geNorm	*α-TUB1* */DnaJ*		*ACT*	*TIP41*	*UBQ*	*β-TUB*	*α-TUB2*	*H2A*	*CDC2*
NormFinder	*ACT*	*UBQ*	*DnaJ*	*α-TUB1*	*β-TUB*	*TIP41*	*α-TUB2*	*H2A*	*CDC2*
Delta CT	*ACT*	*β-TUB*	*α-TUB1*	*DnaJ*	*α-TUB2*	*TIP41*	*UBQ*	*H2A*	*CDC2*
RefFinder	*ACT*	*DnaJ*	*UBQ*	*α-TUB1*	*TIP41*	*β-TUB*	*α-TUB2*	*H2A*	*CDC2*
**Ranking order in Total ( Better–Good–Average)**									
BestKeeper	*UBQ*	*ACT*	*α-TUB2*	*DnaJ*	*TIP41*	*H2A*	*CDC2*	*β-TUB*	*α-TUB1*
geNorm	*ACT* */* *UBQ*		*α-TUB1*	*β-TUB*	*TIP41*	*DnaJ*	*α-TUB2*	*H2A*	*CDC2*
NormFinder	*ACT*	*UBQ*	*DnaJ*	*β-TUB*	*TIP41*	*H2A*	*α-TUB2*	*α-TUB1*	*CDC2*
Delta CT	*ACT*	*UBQ*	*DnaJ*	*β-TUB*	*TIP41*	*α-TUB2*	*α-TUB1*	*H2A*	*CDC2*
RefFinder	*ACT*	*UBQ*	*DnaJ*	*β-TUB*	*TIP41*	*α-TUB2*	*α-TUB1*	*H2A*	*CDC2*

## Data Availability

Data are contained within the article or Supplementary Materials.

## Supplementary Materials

The following supporting information can be downloaded at: https://www.mdpi.com/article/10.3390/cimb46100640/s1. Figure S1: PCR products shown on 1% agarose gels; Figure S2: Single peaks in melting curves for 10 reference genes; Figure S3: Amplification plots of the ten candidate reference genes.
