# Supplementary material for: Identification of the Optimal Quantitative RT-PCR Reference Gene for Paper Mulberry (Broussonetia papyrifera)
Source: Curr Issues Mol Biol. 2024 Sep 26;46(10):10779–94. doi: 10.3390/cimb46100640 (PMC11506246; doi:10.3390/cimb46100640)
Supplement: Supplementary file 1 [file cimb-46-00640-s001.zip › cimb-3181168-supplementary.pdf]

Supplementary Materials

Identification of the Optimal Quantitative RT-PCR Reference Gene for Paper Mulberry (*Broussonetia papyrifera*)

Fangwei Zhou, Liang Xu, Congguang Shi, Fengying Wu and Shaozong Yang *

Zhejiang Key Laboratory of Forest Genetics and Breeding, Zhejiang Academy of Forestry, Hangzhou 310023, China; zhoufangwei@njfu.edu.cn (F.Z.); xuliang@zjforestry.ac.cn (L.X.); shicongguang@zjforestry.ac.cn (C.S.); 18329041683@163.com (F.W.)

* Correspondence: yangshaozong@zjforestry.ac.cn; Tel.: +86-153-7205-8197

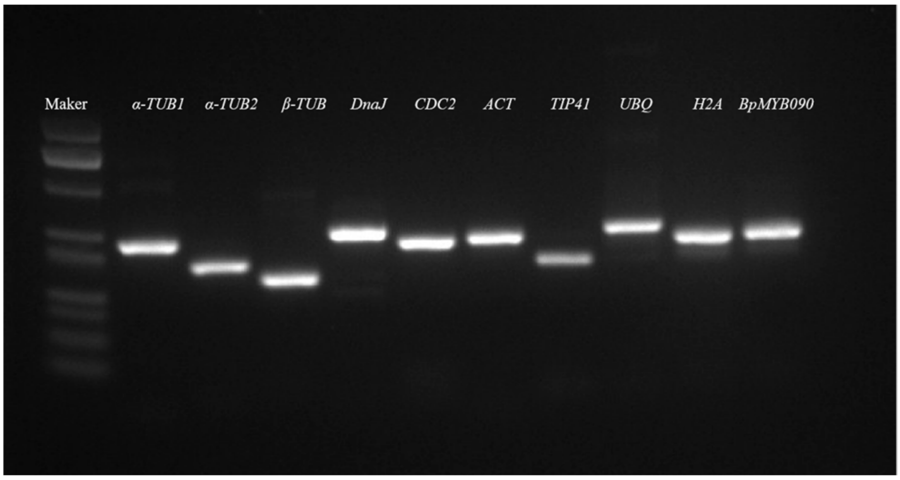

Figure S1. PCR products shown on 1% agarose gels.

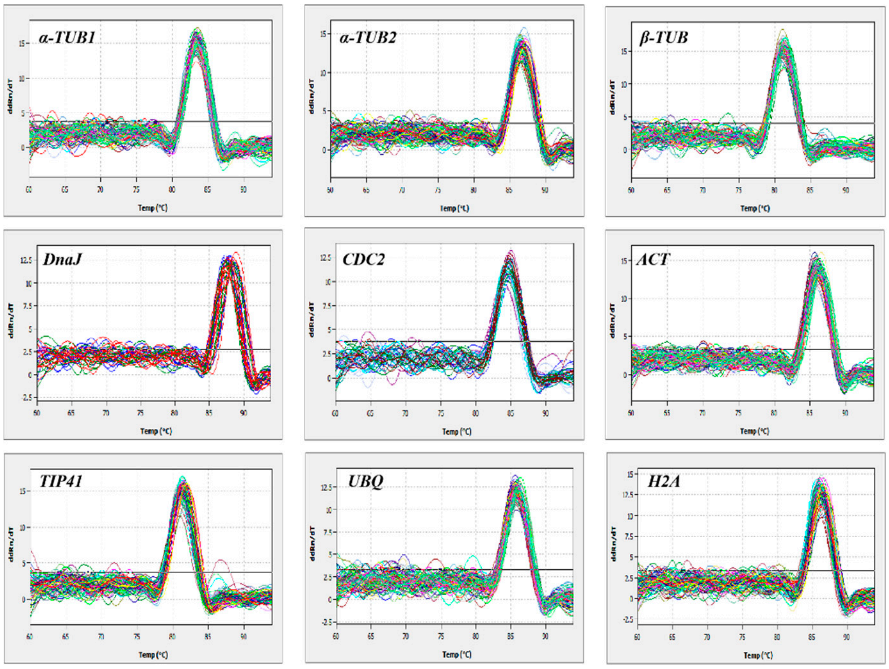

Figure S2. Single peaks in melting curves for 10 reference genes.

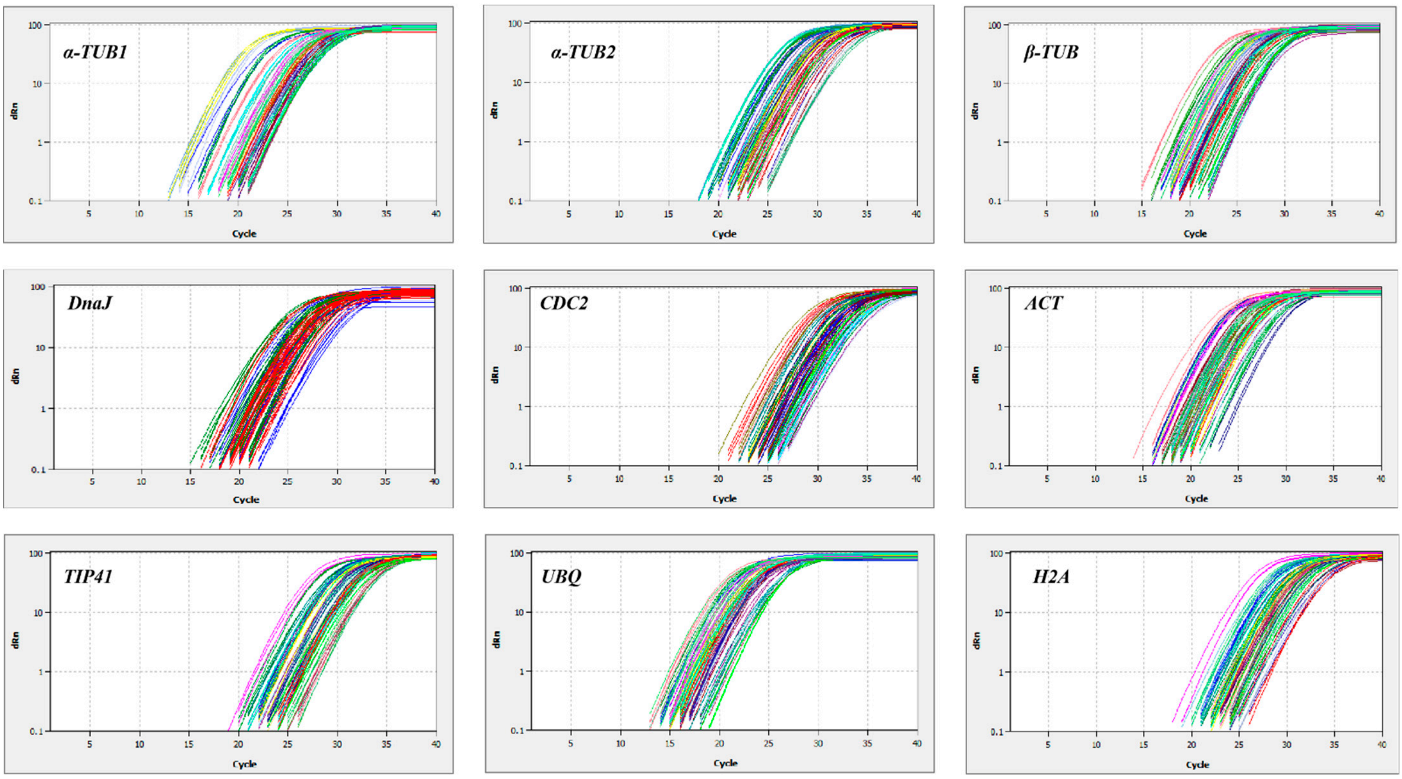

Figure S3. Amplification plots of the ten candidate reference genes.
